# Acute kidney injury in hospitalized patients with nonmalignant pleural effusions: a retrospective cohort study

**DOI:** 10.1186/s12882-024-03556-4

**Published:** 2024-04-01

**Authors:** Danni Wang, Yue Niu, Dinghua Chen, Chaofan Li, Fei Liu, Zhe Feng, Xueying Cao, Li Zhang, Guangyan Cai, Xiangmei Chen, Ping Li

**Affiliations:** 1 Department of Nephrology, First Medical Center of Chinese PLA General Hospital, National Key Laboratory of Kidney Diseases, National Clinical Research Center for Kidney Diseases, Beijing Key Laboratory of Kidney Diseases Research, Beijing, 100853 China; 2 Department of Urology, Chinese Academy of Medical Sciences and Peking Union Medical College, Beijing, China

**Keywords:** Nonmalignant pleural effusion, Acute kidney injury, Risk factor, Prognosis

## Abstract

**Background:**

Nonmalignant pleural effusion (NMPE) is common and remains a definite health care problem. Pleural effusion was supposed to be a risk factor for acute kidney injury (AKI). Incidence of AKI in NMPE patients and whether there is correlation between the size of effusions and AKI is unknown.

**Objective:**

To assess the incidence of AKI in NMPE inpatients and its association with effusion size.

**Study design and method:**

We conducted a retrospective cohort study of inpatients admitted to the Chinese PLA General Hospital with pleural effusion from 2018-2021. All patients with pleural effusions confirmed by chest radiography (CT or X-ray) were included, excluding patients with diagnosis of malignancy, chronic dialysis, end-stage renal disease (ESRD), community-acquired AKI, hospital-acquired AKI before chest radiography, and fewer than two serum creatinine tests during hospitalization. Multivariate logistic regression and LASSO logistic regression models were used to identify risk factors associated with AKI. Subgroup analyses and interaction tests for effusion volume were performed adjusted for the variables selected by LASSO. Causal mediation analysis was used to estimate the mediating effect of heart failure, pneumonia, and eGFR < 60 ml/min/1.73m^2^ on AKI through effusion volume.

**Results:**

NMPE was present in 7.8% of internal medicine inpatients. Of the 3047 patients included, 360 (11.8%) developed AKI during hospitalization. After adjustment by covariates selected by LASSO, moderate and large effusions increased the risk of AKI compared with small effusions (moderate: OR 1.47, 95%CI 1.11-1.94 *p* = 0.006; large: OR 1.86, 95%CI 1.05-3.20 *p* = 0.028). No significant modification effect was observed among age, gender, diabetes, bilateral effusions, and eGFR. Volume of effusions mediated 6.8% (*p* = 0.005), 4.0% (*p* = 0.046) and 4.6% (*p* < 0.001) of the effect of heart failure, pneumonia and low eGFR on the development of AKI respectively.

**Conclusion:**

The incidence of AKI is high among NMPE patients. Moderate and large effusion volume is independently associated with AKI compared to small size. The effusion size acts as a mediator in heart failure, pneumonia, and eGFR.

**Supplementary Information:**

The online version contains supplementary material available at 10.1186/s12882-024-03556-4.

## Introduction

Pleural effusion (PE) is a common clinical manifestation in hospitalized patients and is associated with significant morbidity and mortality. It affects more than 3,000 people per million population each year [1]. Nonmalignant pleural effusions (NMPE) are usually associated with organ dysfunction such as pneumonia and heart failure [2].

Acute kidney injury (AKI) is a common syndrome of complex etiology and pathophysiological mechanisms leading to abrupt loss of renal function. Hospital acquired AKI (HA-AKI) is reported to happen in 9.1% patients that not only associated with short-term adverse outcomes, but also has a long-term negative impact on survival [3–6]. Even mild AKI is considered to be an independent predictor of various adverse outcomes [7, 8].

Large effusions can lead to a number of pathophysiological changes such as hypoxia and hemodynamic compromise putting patients at risk of AKI [9, 10]. What's more, Siniorakis et al. hypothesized a pleurorenal interaction, suggesting that neurohormonal derangements occur in PE patients, leading to renal hemodynamic alterations and AKI [11]. However, the incidence of AKI in hospitalized patients with NMPE is not well understood and there are few data on the relationship between effusion volume and AKI. Therefore, we conducted a large cohort of consecutive patients based on the electronic medical record (EMR) to determine the incidence of AKI in NMPE and its association with the size of effusion in hospitalized NMPE patients.

## Methods

### Study design and population

We conducted a retrospective cohort of inpatients admitted to Chinese People’s Liberation Army (PLA) General Hospital, the largest tertiary care academic hospital in China based on EMR. The EMR in the PLA general hospital is a unified database, with data collected by each department [12]. We included all patients who: 1) were admitted to the internal medicine wards, excluding oncology and hematology departments, between 1 January 2018 and 31 December 2021; 2) diagnosed with pleural effusion by chest CT or X-ray. Patients were excluded if they had: 1) a discharge diagnosis of malignancy such as lung cancer, breast cancer or leukemia; 2) less than two serum creatinine (sCr) tests during hospitalization; 3) end-stage renal disease (ESRD), kidney transplantation or maintenance dialysis. To ensure the temporal relationship between effusion and AKI, we focused on patients with hospital-acquired AKI (HA-AKI) after CT/X-ray exams, excluding HA-AKI before the exams and community-acquired AKI (CA-AKI), which was defined as: 1) patients admitted with AKI according to the diagnosis code; 2) the change of sCr within 24 h after admission met the KDIGO definition [6]. The study was approved by the Research Ethics Committee of the PLA General Hospital (No.S2022-730–01).

### Definition and measurement

The diagnosis of pleural effusion was confirmed by CT and X-ray images, which were read independently by two radiologists. Effusions were quantified by size as small (0–25%), moderate (25–50%) and large (50–100%) at the midclavicular line based on the division of the hemithorax on CT [13]. On the postero-anterior chest radiograph, we classified effusion volume being small as obliteration of the costophrenic angle, moderate as extending from the diaphragm to the pulmonary hilum, and large as extending beyond the hilar region [14].

AKI was defined based on the KDIGO criteria: 0.3 mg/dl increase in sCr or ≥ 1.5–1.9 times baseline as stage 1; sCr ≥ 2–2.9 times baseline as stage 2; sCr > 3 times baseline or ≥ 4.0 mg/dl or RRT therapy during hospitalization as stage 3 [15]. Diagnostic criteria for changes in urine output were not used due to lack of data. The lowest sCr during hospitalization was used as baseline. Estimated glomerular filtration rate (eGFR) was calculated by the CKD-EPI formula with baseline creatinine [16].

### Outcome measures

The primary outcome was the development of hospital-acquired AKI. Outcomes were ascertained from data extracted from the EMR.

### Covariates

Clinical characteristics were obtained from the EMR, including demographics, ICU admission, mechanical ventilation, thoracentesis, medications, comorbidities, and laboratory results. The laboratory results closest to the date of the CT or X-ray examination were recorded. The Charlson comorbidity index was obtained to summarize comorbidity information. Pneumonia was defined as implied pneumonia in chest image with a diagnosis of pneumonia at discharge.

### Statistical analysis

Continuous variables were compared using Student's t-test (for normally distributed variables) or Wilcoxon rank sum test (for non-normally distributed variables). Categorical variables were compared using the chi-squared test. Univariate and multivariate logistic regression was used to determine risk factors associated with the occurrence of HA-AKI. Covariates for multivariate regression models were selected by univariate analysis. Odds ratios (OR), 95% confidence intervals, and *P* values were reported for comparison. To reduce the risk of overfitting in ordinary logistic models and to select the most important predictive factors, we used the LASSO logistic regression model to select the most useful predictive factors for AKI in NMPE patients [17]. We also used multivariate logistic regression, adjusted for the variables selected by LASSO, to explore the effect of effusion volume on different subgroups and estimated the significance of their interactions using likelihood ratio tests. To assess the mediating effect of effusion volume, we used causal mediation analysis to estimate the extent to which effusion volume mediates the effect of heart failure, pneumonia and eGFR below 60 mL/min/1.73 m2 on AKI [18]. We generated three independent single mediator models with volume as the assumed mediator and adjusted each of the three models for age, sex, ICU admission, and use of spironolactone and loop diuretics. We estimated the average causal mediation effect (ACME), the average direct effect (ADE) and the average total effect (ATE). All analyses were performed with R4.2.0. Two-sided nominal *p*-values < 0.05 were considered statistically significant.

## Results

**Fig. 1:**
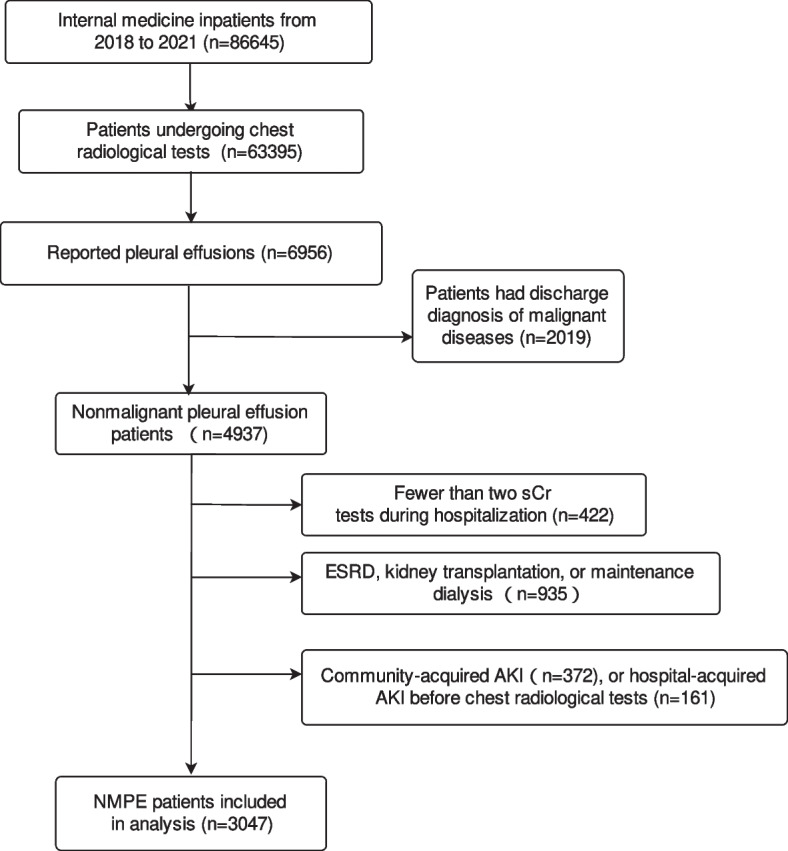
The inclusion flowchart

A total of 86,645 inpatients were screened between January 1, 2018, and December 31, 2021. Among them, 63,395 (73.2%) underwent chest radiological tests (CT/X-ray), and 6,956 of them had pleural effusions. After excluding 2019 cases with a discharge diagnosis of malignant diseases, there were 4937 cases, accounting for 7.8% of those admitted to internal medicine. After excluding 422 cases with sCr tests fewer than 2 during hospitalization, 935 cases diagnosed end-stage renal disease, regular dialysis, or kidney transplantation, 372 cases having CA-AKI and 161 cases having HA-AKI before chest image exams, 3047 cases were included in the final analysis (Fig. 1). Among the 3047 patients, 2019 (66.3%) cases of effusions were diagnosed with CT scan, 924 (30.3%) were diagnosed by orthostatic plain radiograph, and 104 (3.4%) were diagnosed by supine radiograph.

### Incidence of AKI in NMPE inpatients

Of the 3047 patients, a total of 360 (11.8%) developed AKI, of which 232 patients had stage 1, 81 patients had stage 2 and 47 patients had stage 3 AKI. Table 1 shows the demographics, imaging characteristics, treatments, medication, and laboratory results of AKI group and non-AKI group. Patients in the AKI group were older and had lower baseline eGFR than the non-AKI group. The proportion of patients with heart failure (38.3% vs. 61.9%), pneumonia (35.6% vs. 51.7%) and ICU admission (15.4% vs. 38.9%) and the median CCI score (1 vs. 2) were significantly higher in the AKI group. The use of glycopeptide antibiotics, aminoglycoside antibiotics, loop diuretics, vasoactive drugs and digitalis was higher in the AKI group. Patients in the AKI group had higher white blood cell count, C-reactive protein (CRP), lower platelet count and a higher proportion of proteinuria. More patients in the AKI group had moderate or large effusions (moderate 35.3% vs 18%, large 6.4% vs 2.6%).

**Table 1:** The characteristics of NMPE patients and comparison between AKI and non-AKI group

n	Overall	Non-AKI	AKI	p
	3047	2687	360	
eGFR (mean (SD)) (mL/min/1.73m^2^)	84.87 (31.24)	86.90 (30.05)	69.76 (35.54)	< 0.001
Age (median [IQR])	66 [50, 80]	64 [49, 79]	78 [60.75, 87]	< 0.001
Female (%)	1119 (36.7)	977 (36.4)	142 (39.4)	0.279
Bilateral (%)	1907 (62.6)	1685 (62.7)	222 (61.7)	0.744
Pleural effusion volume (%)				< 0.001
Small	2342 (76.9)	2132 (79.3)	210 (58.3)	
Median	612 (20.1)	485 (18.0)	127 (35.3)	
Large	93 (3.1)	70 (2.6)	23 (6.4)	
ICU admission (%)	555 (18.2)	415 (15.4)	140 (38.9)	< 0.001
WBC count (median [IQR]) (*10^9/L)	7.00 [5.35, 9.56]	6.91 [5.30, 9.36]	7.78 [5.79, 11.15]	< 0.001
Serum sodium (median [IQR]) (mmol/L)	139.80 [136.90, 142.20]	139.80 [137.00, 142.10]	139.70 [136.00, 143.10]	0.716
Serum potassium (median [IQR]) (mmol/L)	3.89 [3.56, 4.20]	3.88 [3.56, 4.19]	3.97 [3.57, 4.40]	0.003
Proteinuria (%)	1037 (35.4)	867 (33.6)	170 (48.4)	< 0.001
Platelet count (median [IQR]) (*10^9/L)	201.00 [148.00, 265.00]	205.00 [151.00, 269.00]	172.50 [114.00, 227.00]	< 0.001
CRP (median [IQR]) (mg/L)	0.50 [0.10, 2.50]	0.40 [0.10, 2.30]	1.40 [0.31, 4.70]	< 0.001
Serum albumin (median [IQR]) (g/L)	33.00 [27.90, 37.10]	33.10 [27.90, 37.20]	32.20 [28.10, 36.00]	0.056
Mechanical ventilation (%)	211 (6.9)	142 (5.3)	69 (19.2)	< 0.001
Thoracentesis (%)	212 (7.0)	165 (6.1)	47 (13.1)	< 0.001
Exudates^a^ (n/total (%))	96/177 (54.2)	80/141 (56.7)	16/36 (44.4)	0.186
Contrast agents (%)	671 (22.0)	624 (23.2)	47 (13.1)	< 0.001
NSAIDs (%)	648 (21.3)	543 (20.2)	105 (29.2)	< 0.001
Vancomycin/Teicoplanin (%)	379 (12.4)	285 (10.6)	94 (26.1)	< 0.001
Aminoglycosides (%)	72 (2.4)	57 (2.1)	15 (4.2)	0.027
Spironolactone (%)	1320 (43.3)	1103 (41.0)	217 (60.3)	< 0.001
ACEI/ARB (%)	1124 (36.9)	1008 (37.5)	116 (32.2)	0.058
Loop diuretics (%)	2036 (66.8)	1709 (63.6)	327 (90.8)	< 0.001
Vasoactive agents (%)	233 (7.6)	148 (5.5)	85 (23.6)	< 0.001
Digitalis (%)	382 (12.5)	301 (11.2)	81 (22.5)	< 0.001
Heart failure (%)	1252 (41.1)	1029 (38.3)	223 (61.9)	< 0.001
Pneumonia (%)	1142 (37.5)	956 (35.6)	186 (51.7)	< 0.001
Pancreatitis (%)	90 (3.0)	79 (2.9)	11 (3.1)	1
CCI (median [IQR])	2.00 [1.00, 3.00]	1.00 [1.00, 3.00]	2.00 [1.00, 4.00]	< 0.001

*Abbreviation: eGFR* estimated glomerular filtration rate, *WBC* white blood cell, *CRP* C-reactive protein, *ACEI* Angiotensin-Converting Enzyme Inhibitors, *ARB* angiotension II receptor blocker, *NSAIDs* nonsteroidal anti-inflammatory drug, *CCI* Charlson Comorbidity Index

^a^Effusions were classified either as transudative or exudative by Light’s criteria

### Risk factors for HA-AKI among NMPE patients

**Fig. 2:**
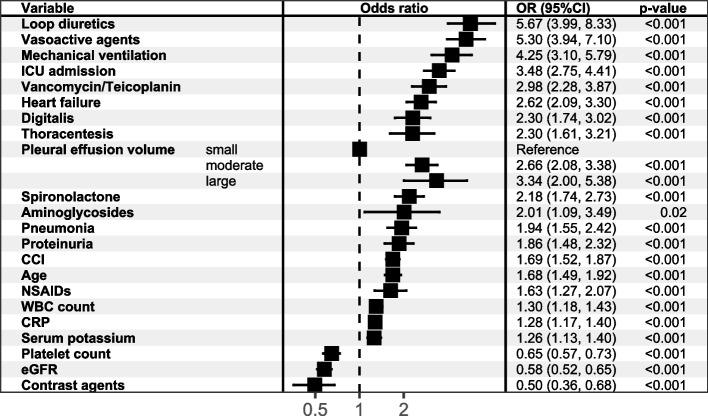
The results of univariate logistic regressions modeling HA-AKI in NMPE patients. Abbreviation: eGFR = estimated glomerular filtration rate; WBC = white blood cell; CRP = C-reactive protein; NSAIDs = non-steroidal anti-inflammatory drug; CCI = Charlson Comorbidity Index

**Fig. 3:**
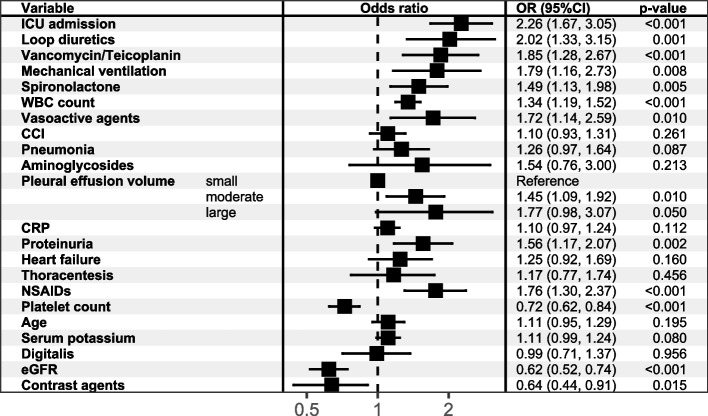
The result of multivariate logistic regressions modeling HA-AKI in NMPE patients. Abbreviation: eGFR = estimated glomerular filtration rate; WBC = white blood cell; CRP = C-reactive protein; NSAIDs = non-steroidal anti-inflammatory drug; CCI = Charlson Comorbidity Index

The result of univariate logistic regression is shown in Fig. 2. Multivariate logistic regression showed that in addition to volume of effusions, mechanical ventilation, ICU admission, use of vancomycin/teicoplanin, vasoactive drugs, spironolactone, loop diuretics, NSAIDs, low eGFR and platelets, high leukocyte count, proteinuria were independent risk factors for AKI during hospitalization (Fig. 3).

**Fig. 4:**
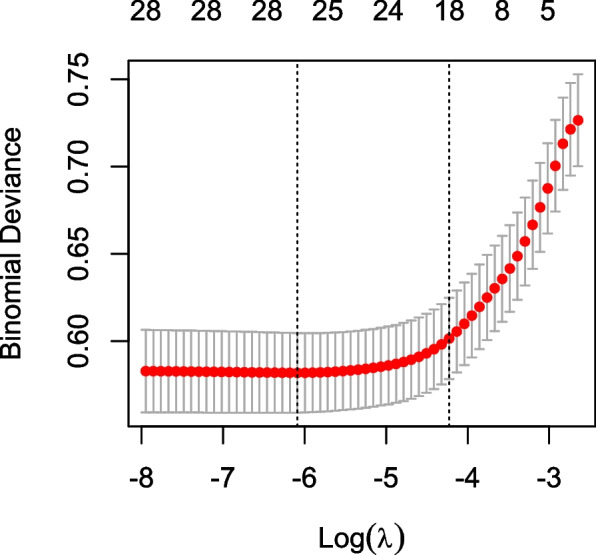
The cross-validation results. The value between the two dotted lines is the range of the standard deviations of log (λ). The right vertical line indicated the value of log (λ) when the error of the model is minimized

**Fig. 5:**
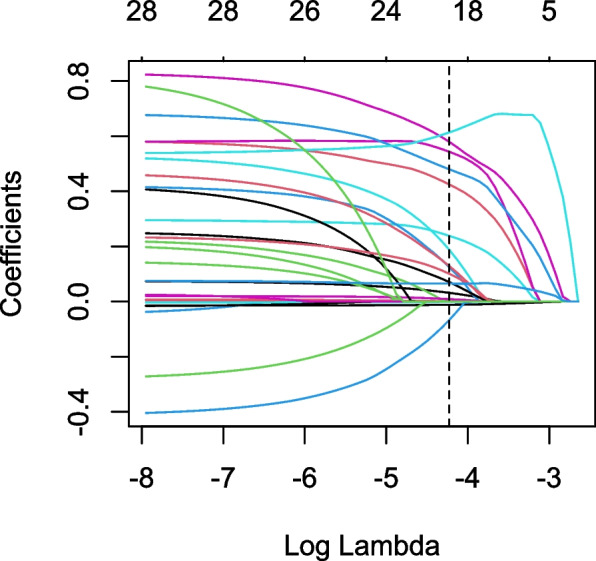
LASSO coefficient profiles of the variables. A vertical line was drawn at the value chosen by cross-validation. As the value of λ decreased, the degree of model compression increased and the function of the model to select important variables increased

**Fig. 6:**
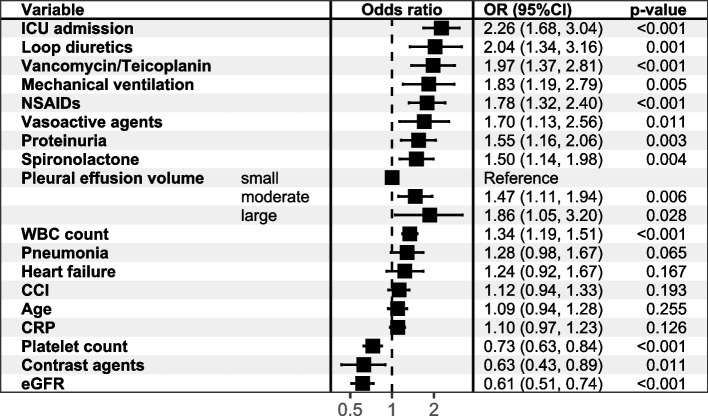
Risk factors selected by LASSO logistic regression model. 18 variables were selected with their odds ratio, 95%CI and p value shown

Then, we used LASSO regression to select variables. The number of identified variables selected by cross-validation is shown in Fig. 4. The LASSO coefficient profiles of the 28 variables and the result of selection by threshold are shown in Fig. 5. After LASSO logistic analysis, 18 risk factors were selected, including age, pleural effusion volume, ICU admission, eGFR, WBC count, proteinuria, platelet count, CRP, mechanical ventilation, contrast agents, NSAIDs, vancomycin/teicoplanin, spironolactone, loop diuretics, vasoactive agents, heart failure, pneumonia and CCI (Fig. 6).

### Association between pleural effusion volume and HA-AKI in NMPE patients

Patients with moderate and large effusion volume were 2.66 (*p* < 0.001) and 3.34 (*p* < 0.001) times more likely to develop AKI, respectively, compared with small effusions in univariate analysis. After adjustment by covariates selected by LASSO, moderate and large effusions increased the odds of AKI compared with small effusions (moderate: OR 1.47, 95% CI 1.11–1.94 *p* = 0.006; large: OR 1.86, 95% CI 1.05–3.20 *p* = 0.028).

### The effect of volume of effusions on different subgroup

**Fig. 7:**
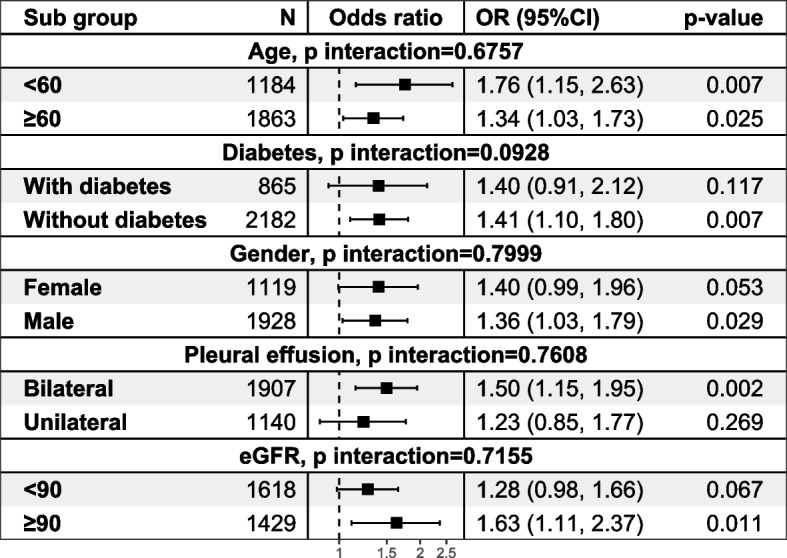
Subgroup analysis and interaction tests for effusion volume. Each model was adjusted for the 18 variables selected by LASSO regression. The likelihood ratio test in each subgroup shows no statistical significance

The forest plot of effusion volume in different subgroups is shown in Fig. 7. We found that effusion volume was associated with increased odds of AKI in male patients (OR = 1.36, 95% CI 1.03–1.79), patients without diabetes (OR = 1.41, 95%CI 1.10–1.80), patients with bilateral effusions (OR = 1.50, 95%CI 1.15–1.95), patients with eGFR above 90 mL/min/1.73 m2 (OR = 1.63, 95%CI 1.11–2.37). The size of effusions increased the odds more in patients under 60 years of age (OR = 1.76, 95%CI 1.15–2.63) compared to patients over 60 years of age (OR = 1.34, 95%CI 1.03–1.73). The likelihood ratio test in each subgroup gives a p-value ranging from 0.0928 to 0.7999, indicating that there are no significant interactions between effusion volume and the indicator variables.

### Causal mediation analysis of volume of effusions

**Fig. 8:**
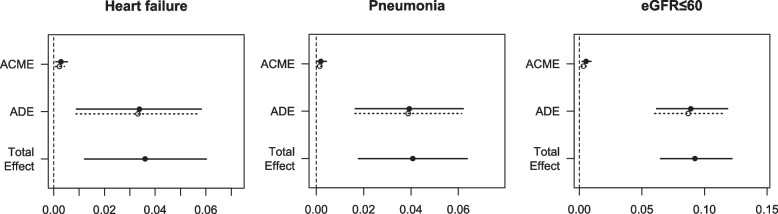
Effect decomposition plots for each mediator model. ACME average causal mediation effect, ADE average direct effect. The total effect is shown as the average effect. All effects are shown with their 95% confidence intervals

Mediation analyses showed that effusion volume might work as a mediator of heart failure, pneumonia and low eGFR on the development of AKI, with ACMEs 0.0025, 0.0017, 0.0043, respectively. The ACMEs are significant, although most of the effect of the three variables couldn't be explained by effusion volume, with the proportion of effect mediated being 6.8% (*p* = 0.005), 4.0% (*p* = 0.046) and 4.6% (*p* < 0.001) respectively. The decompositions of the effects are shown in Fig. 8.

## Discussion

Our results showed that of the 3047 patients, 360 (11.8%) developed AKI during hospitalization. Although malignant pleural effusions usually accompanied with poor outcome, worse prognosis in patients with NMPE due to organ dysfunction was being noticed [19]. AKI should be considered as a systemic problem leading to multi-organ injury and even mild AKI is related to short or long adverse events. Thus, AKI might be crucial to be prevented and treated to improve NMPE patients’ prognosis.

We hypothesized that the volume of effusions related to AKI, which was verified in multivariate logistic regression after adjusting all the covariates we thought related to AKI. Large pleural effusions can alter respiratory mechanics and lead to abnormal gas exchange, reduction in diaphragmatic function or pleural irritation. Animal models have been used to study the effects of varying pleural effusion volumes and have shown that increased pleural effusion volume is associated with decreased PaO2, increased intrapulmonary shunts and a significant decrease in left ventricular preload [20–22]. These have been verified in human patients. Razazi K et al. reported that drainage of large pleural effusions improved oxygenation and end-expiratory lung volume [9]. Another prospective study showed improved preload, systolic and diastolic function and hemodynamic changes after drainage of large effusions [10]. What's more, in a case report of a patient with acute kidney injury in the course of a large pleural effusion, the authors suggested an interdependence between pleural effusion and kidney injury, which was attributed to neurohormonal derangements, affecting the secretion of natriuretic peptides and/or antidiuretic hormone [11]. In another study of patients having non-cardiac pleural effusion with normal blood pressure and renal function, the renal artery resistance index (RI) and pulsatility index (PI) of patients having effusion were higher than those normal controls [23]. The above hypotheses, suggesting that pleural effusion may lead to complicated pathophysiological changes in kidney through neurohumoral regulation, are proposed by clinical manifestations and rationality needs further verification. We also need consider that pleural effusion is one of the prognostic scores of pneumonia and acute pancreatitis [24, 25], and pleural effusion volume is associated with poor outcomes in these acute conditions [26, 27]. For non-specific conditions, a prospective multicenter study of hospitalized patients with PE showed that large effusions were associated with higher mortality than small effusions at 1 year after adjustment for other factors [13]. AKI was known to be a measurable prognostic marker in several types of disease, as demonstrated by many studies [28–30]. Therefore, size of effusions could be expected to be related to AKI for their common association with disease severity which can’t be fully adjusted.

A meta-analysis showed that older age was associated with a higher risk of AKI, but this effect was attenuated by lower eGFR or higher albumin-to-creatinine ratio (ACR), and eGFR and ACR were consistent, strong risk factors for AKI [31]. In our study, the odds ratio of age was 1.68 (95%CI 1.49–1.92; *p* < 0.001) in univariate analysis, but in multivariate analysis and LASSO analysis, the odds ratio of age was attenuated with no statistically significant difference, although it was selected by LASSO analysis, which might be due to other stronger risk factors. Proteinuria and eGFR remained independent risk factors in our study. 37.5% of patients had pneumonia, so infection was a very common problem in NMPE patients if abdominal and intracranial infection were added. We found vancomycin/teicoplanin was an independent contributor to AKI with a relatively high odds ratio (OR = 1.97, 95% CI 1.38–2.81). The exact mechanism by which vancomycin/teicoplanin induces nephrotoxicity is unclear, which was reported to be associated with oxidative stress [32]. Although vancomycin/teicoplanin is not the preferred anti-infective agent because of its side effects, it is one of the few antibiotics of choice for methicillin-resistant Staphylococcus aureus (MRSA) infections. Clinicians should carefully monitor urine output and creatinine when using vancomycin/teicoplanin. Our results suggest that both spironolactone and loop diuretics are risk factors for AKI in multivariate analysis with a relative high odds ratio. This may be related to concomitant heart failure, renal insufficiency and cirrhosis, or reduced renal perfusion with diuretics. Therefore, a better way to use diuretics in NMPE patients needs to be further investigated. In addition, the use of NSAIDs was also an independent risk factor for HA-AKI with a high odds ratio, which may be related to the hemodynamic changes or damage to the renal interstitium from NSAIDs [33]. In addition, uncontrolled infection causing fever or rapid volume depletion due to diaphoresis after NSAIDs use could be confounders, so NMPE patients may be at high risk when using NSAIDs and should be used cautiously or in reduced doses when it's necessary to improve symptoms. The Charlson comorbidity index (CCI) was a predictor of AKI in many diseases [34–36]. Previous studies showed that the number of comorbidities and CCI were predictors of both short-term and long-term mortality in patients with PE [13, 37]. We hypothesized that NMPE patients hospitalized with multiple comorbidities may have had an acute change in condition during admission leading to acute kidney injury, considering a high burden of comorbidity was quite common in NMPE patients (the median CCI was 2 in our study and 5 in the previous research) [13]. The odds ratio of CCI was 1.69 in our univariate analysis (95%CI 1.52–1.87), but was not significant after adjustment for other variables. We found that effusion volume might work as a mediator between heart failure, pneumonia or eGFR and AKI, although the effect was small. This may support our hypothesis that the volume of PE was associated with AKI. Researches have shown that in the course of progression of pneumonia-related pleural effusion, imbalance of various pro- and anti-inflammatory factors, abnormal activity of the fibrinolytic system play an important role, which have been considered as mechanisms for the development of AKI in a variety of models [38–40] as well. Thus, there may be a common pathway for the development of AKI and increase of pleural effusion. Besides, we hypothesized that the mechanism of the mediating effect of effusions on heart failure and eGFR might be hemodynamic changes. The above findings of an underlying mediating effect of volume can only be considered hypothesis-generating, showing possible associations but not necessarily causal relationships. It cannot be excluded that similar changes obtained by other approaches than the volume of effusions may contribute.

In fact, in the majority of cases, clinicians couldn't determine the course of the effusion because of its insidious onset. Meanwhile, most patients had more than one etiology and only a few of them had fluid examination results [37]. Thus, much remains to be done. Our study found AKI incidence was high among NMPE patients and analyzed the risk factors and the correlation between the size of effusion and AKI, attempting to provide some implications for clinicians to improve the prognosis of the underlying high mortality patients through the development of AKI. However, it has some limitations. First, measurement bias was likely because supine radiographs are only moderately sensitive and specific for assessing pleural effusions, but it only accounted for 3.4% in our study and we included these patients to get as close to the real scenario as possible. Second, due to the retrospective nature of the study, only a proportion of patients had urine output data, which may lead to an underestimation of the incidence of AKI grade [41]. In addition, we are unable to assess the fluid balance status of patients because of the lack of reliable data such as central venous pressure (CVP). Some important risk factors such as shock and sepsis might be missed, and instead, we included vasoactive drugs (used before AKI) as covariates in the multiple model. Third, we didn't follow up patients to get long-term outcomes. Forth, the outcome in our study was unbalanced, which might introduce sparse effects [42]. To evaluate this effect, we used Firth-type penalization and there was no significant difference between original multivariate logistic regression and Firth’s logistic regression model (see Additional file 1). Finally, this was a single-center study and there may be unmeasured confounders, so the causal relationship needs future studies supported by a larger sample from multiple centers.

## Conclusion

The incidence of AKI is high among NMPE patients and large effusion volume is independent associated with AKI compared to small size. The effusion size acts as a mediator in heart failure, pneumonia, and eGFR.

### Supplementary Information

**Additional file 1.** Comparison of multivariate logistic and firth logistic regression. The two models are almost identical and the sparse effect might be negligible.

## Data Availability

Some or all datasets generated during and/or analyzed during the current study are not publicly available but are available from the corresponding author upon reasonable request.

## Abbreviations

AKI: Acute kidney injury
EMR: Electronic medical record
CI: Confidence interval
eGFR: Estimated glomerular filtration rate
IQR: Interquartile range
NMPE: Nonmalignant pleural effusion
NT-proBNP: N-terminal pro-B-type natriuretic peptide
SD: Standard deviation
PE: Pleural effusion
