# Supplementary material for: Acute kidney injury in hospitalized patients with nonmalignant pleural effusions: a retrospective cohort study
Source: BMC Nephrol. 2024 Apr 1;25:118. doi: 10.1186/s12882-024-03556-4 (PMC10983765; doi:10.1186/s12882-024-03556-4)
Supplement: Supplementary file 1 — Additional file 1. Comparison of multivariate logistic and firth logistic regression. The two models are almost identical and the sparse effect might be negligible. [file 12882_2024_3556_MOESM1_ESM.docx]

Supplementary table： Comparison of multivariate logistic regression and firth logistic regression. The two models are almost identical and the sparse effect might be negligible.

|  | Original logistic model | | Firth logistic model | |
| --- | --- | --- | --- | --- |
|  | coef | se(coef) | coef | se(coef) |
| (Intercept) | -4.07261 | 0.221068 | -4.01929 | 0.216347 |
| Loop diuretics | 0.704718 | 0.219025 | 0.69436 | 0.21451 |
| Vasoactive agents | 0.542021 | 0.209693 | 0.533572 | 0.206186 |
| Mechanical ventilation | 0.580843 | 0.217401 | 0.573281 | 0.213566 |
| ICU admission | 0.816812 | 0.153293 | 0.806662 | 0.150869 |
| Vancomycin/Teicoplanin | 0.617423 | 0.187312 | 0.610137 | 0.184156 |
| Heart failure | 0.219768 | 0.156448 | 0.214376 | 0.15393 |
| Digitalis | -0.00924 | 0.168886 | -0.00663 | 0.166259 |
| Thoracentesis | 0.154996 | 0.207701 | 0.159784 | 0.204009 |
| Pleural effusion volume: moderate | 0.371705 | 0.143501 | 0.369889 | 0.141384 |
| Pleural effusion volume: large | 0.568238 | 0.289495 | 0.573617 | 0.28328 |
| Spironolactone | 0.401171 | 0.142454 | 0.395547 | 0.14024 |
| Aminoglycosides | 0.43489 | 0.349529 | 0.44945 | 0.340335 |
| Pneumonia | 0.232378 | 0.135585 | 0.230462 | 0.133414 |
| Proteinuria | 0.444279 | 0.145329 | 0.43714 | 0.143068 |
| CCI | 0.099196 | 0.088192 | 0.100509 | 0.086753 |
| Age | 0.102244 | 0.078851 | 0.099367 | 0.077535 |
| NSAIDs | 0.565104 | 0.152881 | 0.560344 | 0.150338 |
| WBC count | 0.296138 | 0.062353 | 0.291725 | 0.061145 |
| CRP | 0.097113 | 0.061159 | 0.099485 | 0.059625 |
| Serum potassium | 0.101708 | 0.058147 | 0.10095 | 0.057211 |
| Platelet count | -0.32341 | 0.075766 | -0.31769 | 0.074357 |
| eGFR | -0.47841 | 0.09352 | -0.46931 | 0.091924 |
| Contrast agents | -0.45109 | 0.184673 | -0.43868 | 0.18112 |
